# The use of cannabidiol (CBD) as an analgesic component

**DOI:** 10.1016/j.lanepe.2023.100791

**Published:** 2023-11-14

**Authors:** Kristian Kjær-Staal Petersen, Andrew S.C. Rice, Lars Arendt-Nielsen

**Affiliations:** a Faculty of Medicine, Department of Health Science and Technology, Center for Neuroplasticity and Pain, Aalborg University, Aalborg, Denmark; b Department of Materials and Production, Center for Mathematical Modeling of Knee Osteoarthritis (MathKOA), Aalborg University, Aalborg, Denmark; c Pain Research, Department of Surgery and Cancer, Faculty of Medicine, Imperial College London, London, United Kingdom; d Mech-Sense, Department of Gastroenterology and Hepatology, Aalborg University Hospital, Aalborg, Denmark; e Steno Diabetes Center North Denmark, Clinical Institute, Aalborg University Hospital, Aalborg, Denmark

The use of cannabidiol (CBD) has received growing attention, and cannabis-based medicines are being explored as analgesics. In 2021, a task force, initiated by the International Association for the Study on Pain (IASP), published a series of narrative and systematic reviews and meta-analyses covering multiple aspects of pre-clinical and clinical pain research examining the efficacy and safety of cannabinoids as analgesics. It was concluded that the available evidence does not support the use of cannabinoids as analgesics on either safety or efficacy grounds.^1^ In particular, a meta-analysis of 36 randomised controlled clinical trials (RCTs), which included 7217 subjects, was unable to identify any clinically important analgesic effects, and identified that the current literature was of low and very-low quality.^2^ Since then, 16 RCTs on CBD have been published^3^ but these studies have been criticised for being small or used to low a dose of CBD. High-quality CBD RCTs have therefore been warranted.

In the current issue of *The Lancet Regional Health—Europe*, Pramhas et al.,^4^ investigated, in a high-quality well-designed single-centered RCT in Austria, if a high-dose of oral CBD (600 mg/daily, n = 43) administered over 8 weeks period as an add-on therapy to paracetamol (3 g/daily) could provide an analgesic effect to patients with knee osteoarthritis when compared to placebo and paracetamol (n = 43).

The primary result from the study by Pramhas et al.,^4^ is that 8 weeks of high-dose CBD as an add-on therapy to paracetamol do not provide an analgesic effect when compared to placebo in patients with knee osteoarthritis. Secondary findings included changes in physical function, stiffness of the knee, patient global assessment of change and functional tests, which were all non-significantly different when comparing the CBD and placebo groups. This aligns with four high-quality RCTs within chronic musculoskeletal pain, which have demonstrated no analgesic when studying patients with hand osteoarthritis and psoriatic arthritis, acute non-traumatic low back pain, and postoperative pain after total knee arthroplasty and arthroscopic rotator cuff repair surgery.^3^ Only a single small (n = 18) study has demonstrated an analgesic effect in patients with thumb basal joint arthritis.^3^ Based on the current evidence, the conclusions from the IASP Task Force, and the 17 RCTs, including the new CBD study by Pramhas et al.,^4^ are that CBD cannot be recommended for managing painful musculoskeletal conditions like osteoarthritis.

An important exploratory outcome from the study by Pramhas et al.,^4^ is that the authors sub-grouped patients in responders and non-responders (based on a criterion of a 30% and 50% analgesic effect) to the interventions. In general, long-term pharmacological therapies for musculoskeletal conditions provide an average analgesic effect of 25–30%, but sub-grouping patients into responders and non-responders can tease out potential subsets of patients who might be more likely to respond.^5^^,^^6^ Pramhas et al.,^4^ found no statistical differences in number of responders and non-responders in the 30% and 50% groups, respectively when comparing CBD to placebo. In pain research, the placebo effect is generally large and a recent meta-analysis concluded that the placebo responses contribute significantly to pain reduction in cannabinoid clinical trials, which might be due to the unusually high media attention surrounding cannabinoid trials.^7^

The strength of the study by Pramhas et al.,^4^ is that the trial is sufficiently powered and that it investigates a high dose of CBD, comparable to the doses utilized in previous positive clinical trials for epilepsy. One limitation could be that the study by Pramhas et al.,^4^ is still relatively small and therefore does not allow for sufficient exploratory analysis into subsets of patients who might benefit from CBD. Pre-clinical data suggests a potential analgesic and anti-inflammatory effects of CBD.^8^ The study of Prahmas et al.,^4^ did not assess mechanistic-based pain biomarkers, which would have allowed for better translation between pre-clinical data and clinical data, and this could be viewed as a limitation although the clinical effect on pain is the relevant patient centered outcome. Despite this, the study by Pramhas et al.,^4^ is well-conducted and further indicates that there is likely no place for CBD in pain management of patients with chronic musculoskeletal pain.

Chronic musculoskeletal pain is complex and multiple factors are generally associated with high levels of clinical pain, such as poor quality of sleep, inflammation, psychological factors and sensitization of the nervous system.^9^ Pre-clinical evidence suggests that CBD might target some of these factors^8^ and this could potentially lead to defining a subset of patients who could benefit from CBD. The current evidence from human trials, have demonstrated no effects on neither inflammation nor quality of sleep when comparing CBD to placebo,^10^ but future studies could investigate, in enriched RCTs, whether CBD could potentially have a role in a subset of patients with chronic musculoskeletal pain.

## Contributors

Kristian K-S Petersen received the invitation to write this commentary and wrote the first draft of the manuscript. Lars Arendt-Nielsen and Andrew SC Rice have completed a series of systematic reviews, meta-analyses and recommendations within the area of cannabis-based medicine and provide critical feedback to the manuscript. All authors agreed to submit the final manuscript.

## Declaration of interests

Kristian K-S Petersen chairs the Special Interest Group for Musculoskeletal Pain under the International Association for the study of Pain (IASP). Lars Arendt-Nielsen was the president for the IASP and initiated the IASP Presidential Task Force on Cannabis and Cannabinoid Analgesia and Andrew SC Rice chaired the Task Force.
